# Crystal structure of a calcium(II)–pyrrolo­quinoline quinone (PQQ) complex outside a protein environment^1^

**DOI:** 10.1107/S2053229620014278

**Published:** 2020-11-05

**Authors:** Henning Lumpe, Peter Mayer, Lena J. Daumann

**Affiliations:** a Department of Chemistry, Ludwig-Maximilians-Universität München, Butenandtstrasse 5-13, Munich, Bavaria 81377, Germany

**Keywords:** pyrrolo­quinoline quinone, calcium, PQQ, methanol de­hydrogenase, crystal structure

## Abstract

Pyrrolo­quinoline quinone (PQQ) is an important cofactor of calcium- and lanthanide-dependent alcohol de­hydrogenases, and has been known for over 30 years. Crystal structures of Ca–MDH enzymes (MDH is methanol de­hydrogenase) have been known for some time; however, crystal structures of PQQ with biorelevant metal ions have been lacking in the literature for decades. We report here the first crystal structure analysis of a Ca–PQQ com­plex outside the protein environment, namely, poly[[undeca­aqua­bis­(μ-4,5-dioxo-4,5-di­hydro-1*H*-pyrrolo­[2,3-*f*]quinoline-2,7,9-tri­carboxyl­ato)tricalcium(II)] dihydrate], {[Ca_3_(C_14_H_3_N_2_O_8_)_2_(H_2_O)_11_]·2H_2_O}_*n*_. The com­plex crystallized as Ca_3_PQQ_2_·13H_2_O with Ca^2+^ in three different positions and PQQ^3−^, including an extensive hydrogen-bond network. Similarities and differences to the recently reported structure with biorelevant europium (Eu_2_PQQ_2_) are discussed.

## Abstract 2

Pyrrolo­quinoline quinone (PQQ) is an important cofactor of calcium- and lanthanide-dependent alcohol de­hydrogenases. The crystal structure of a Ca–PQQ com­plex (Ca_3_PQQ_2_·13H_2_O) is reported for the first time outside a protein environment.

## Introduction   

**Figure 1:**
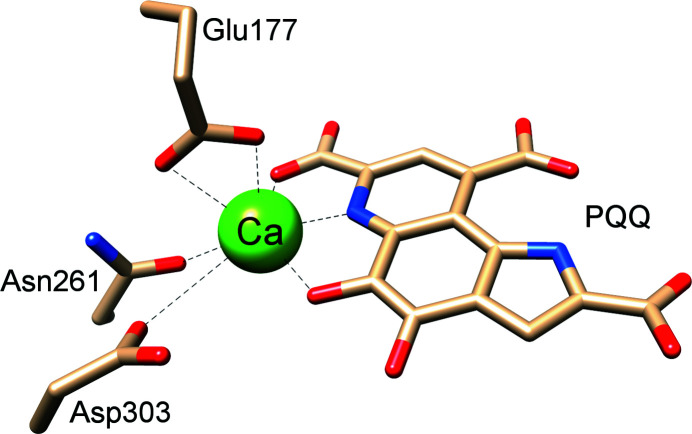
The structure of the active site from Ca-dependent MDH (PDB code 1w6s).

Pyrrolo­quinoline quinone (PQQ) is the redox cofactor of glucose de­hydrogenase enzymes and alcohol de­hydrogenases. In particular, the methanol de­hydrogenase (MDH) enzymes, which catalyze the oxidation of methanol for the energy household of many methano- and methyl­otrophic microorganisms, have attracted attention recently. For proper functionality, a metal ion is needed, which acts as a Lewis acid and which is coordinated by PQQ and several amino acids in the enzymatic active site (Fig. 1 ▸). The Ca-dependent MDH, encoded by the mxaF gene, was first discovered by Anthony & Zatman (1964*a* ▸,*b* ▸).

**Figure 2:**
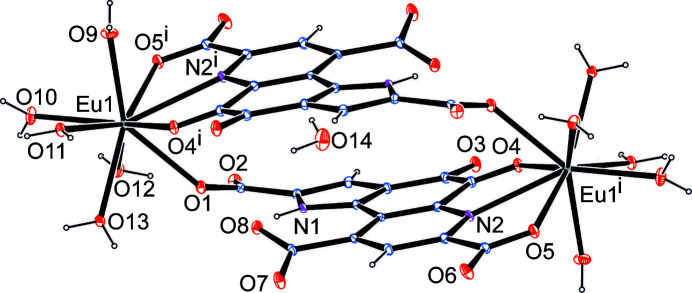
The crystal structure of the inversion-symmetric Eu_2_PQQ_2_ com­plex. The CIF is taken from Lumpe *et al.* (2020 ▸). Displacement ellipsoids are drawn at the 50% probability level. [Symmetry code: (i) −*x* + 1, −*y* + 1, −*z* + 1.]

After the structure of MDH was elucidated in 1978–79 (Duine *et al.*, 1978 ▸; Westerling *et al.*, 1979 ▸; Salisbury *et al.*, 1979 ▸), the cofactor attracted much attention in the following years, with articles published concerning its total synthesis (Corey & Tramontano, 1981 ▸), redox chemistry (Eckert *et al.*, 1982 ▸), metal coordination (Noar *et al.*, 1985 ▸) and small-mol­ecule inter­action (van Koningsveld *et al.*, 1985 ▸). Itoh and co-workers published several articles presenting the inter­action of PQQ with Ca and other alkaline earth metals (Itoh *et al.*, 1997 ▸, 1998 ▸), and the synthesis of model com­pounds, mimicking the active site of MDH (Itoh *et al.*, 2000 ▸). Those publications contributed to a better understanding of the functionality and reactivity of PQQ. However, no crystal structures were presented in those studies, which would reveal in-depth structural information of PQQ–metal inter­actions. While no Ca–PQQ structure has been published to date, in addition, few other crystal structures exist for PQQ with other metals. Outside of the Ca–MDH network (Blake *et al.*, 1994 ▸; Williams *et al.*, 2005 ▸), several structures were published with sodium (Ishida *et al.*, 1989 ▸; Ikemoto *et al.*, 2012 ▸; Ikemoto *et al.*, 2017 ▸), with PQQ structural analogs and iron (Tommasi *et al.*, 1995 ▸), with copper and terpyridine (terpy) as co-ligand (Nakamura *et al.*, 1994 ▸), with copper and tri­phenyl­phosphine (Wanner *et al.*, 1999 ▸), with ruthenium and terpy (Mitome *et al.*, 2015 ▸), and with ruthenium, silver and terpy (Mitome *et al.*, 2013 ▸). In 2014, Pol *et al.* reported a new kind of MDH, found in the extremo­phile *Methyl­acidiphilum fumariolicum SolV* (SolV), which is native to volcanic mudpots close to the Solfatara crater in Italy (Pol *et al.*, 2014 ▸). This MDH turned out to be strictly dependent on lanthanides (Pol *et al.*, 2014 ▸; Lumpe *et al.*, 2018 ▸; Bogart *et al.*, 2015 ▸). While SolV was originally thought to be a biological curiosity, more and more organisms in all kinds of ecosystems were found to be lanthanide dependent in the following years, not restricted to such extreme environments like SolV (Keltjens *et al.*, 2014 ▸; Ramachandran & Walsh, 2015 ▸; Taubert *et al.*, 2015 ▸). This also pushed lanthanide bioinorganic chemistry as a new and emerging scientific field with several reviews published (Skovran & Martinez-Gomez, 2015 ▸; Cheisson & Schelter, 2019 ▸; Chistoserdova, 2019 ▸; Cotruvo, 2019 ▸; Daumann, 2019 ▸; Picone & Op den Camp, 2019 ▸; Semrau *et al.*, 2018 ▸). Recently, also, the first crystal structure of a europium–PQQ com­plex outside the MDH network was published through a collaborative effort and was reported as an Eu_2_PQQ_2_ structure (Lumpe *et al.*, 2020 ▸) (Fig. 2 ▸). In light of those advances and the still scarce structural information available about PQQ–metal inter­actions, we present here the first crystal structure of a Ca–PQQ com­plex without the need of structural PQQ analogs or additional co-ligands. The mol­ecular formula of the com­plex is Ca_3_PQQ_2_·13H_2_O.

## Experimental   

### Materials   

CaCl_2_·2H_2_O (99%) was purchased from VWR. Na_2_PQQ·H_2_O was extracted from Doctor’s Best Science-Based Nutrition BioPQQ capsules, as described previously (Lumpe & Daumann, 2019 ▸). Milli-Q-grade water (pH 5.5), obtained from a Millipore Synergy UV system from Merck (Darmstadt, Germany), was used for all experiments.

### Crystal growth and analysis   

**Figure 3:**
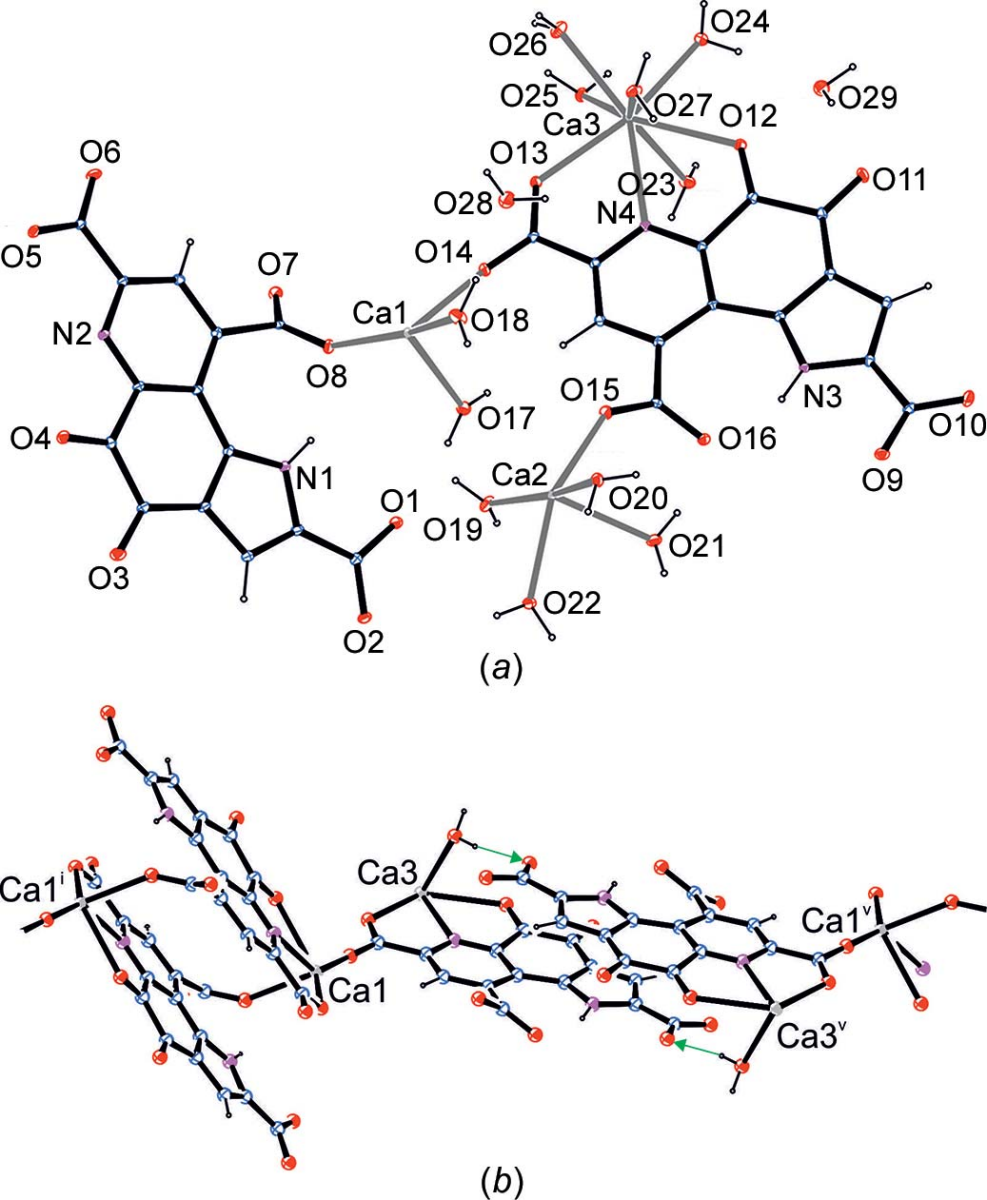
(*a*) The asymmetric unit of Ca_3_PQQ_2_·13H_2_O. (*b*) A strand along [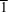
11] consisting of inversion-symmetric Ca_2_PQQ_2_^2−^ pairs. Here, for clarity, all water mol­ecules, except for that involved in intra-pair hydrogen bonds (green arrows), have been omitted. For symmetry codes, see Table 2 ▸.

Na_2_PQQ·H_2_O (32.8 mg, 0.08 mmol) was dissolved in H_2_O (12 ml). CaCl_2_·2H_2_O (2.0 equiv., 23.6 mg, 0.16 mmol) was added as a solid. The metal addition led to precipitation of a pale-grey–brown solid, which was centrifuged, removed and analyzed as a 1:1 PQQ–Ca com­plex, as described in our previous article (Lumpe & Daumann, 2019 ▸). From the supernatant, consisting of a highly diluted aqueous mixture of Na_2_PQQ and CaCl_2_, small dark crystals, suitable for X-ray crystallography, grew over a period of several months. To obtain more crystalline material of better quality, a procedure from our recent publication (Lumpe *et al.*, 2020 ▸) was implemented. Na_2_PQQ·H_2_O (24.2 mg, 61.8 µmol) was com­pletely dissolved in H_2_O (4 ml) at 80 °C in an ultrasonic bath. CaCl_2_·2H_2_O (27.3 mg, 185.4 µmol, 3 equiv.) was dissolved in a small amount of water (0.2 ml) and was added to the Na_2_PQQ solution at 80 °C, which caused precipitation of a grey–brown solid. The mixture was placed directly in a drying oven at 80 °C, which was then switched off and the reaction mixture allowed to cool slowly. After 1 d, small dark crystals had grown between the bulk precipitate. The crystals grew in size over the next few days while consuming the surrounding bulk precipitate. Crystals suitable for X-ray diffraction analysis were then picked out of the reaction mixture. The crystal used for analysis was selected in paraffin oil to prevent dehydration and then placed and measured on a Mitegen Microloop. The crystals obtained from both methods showed the same structure depicted in Fig. 3 ▸.

IR (diamond ATR, neat): 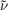/cm^−1^ 3643–2746 (*w*, broad), 1923–1714 (*w*, broad), 1686 (*w*), 1658 (*w*), 1605 (*s*), 1577 (*m*), 1553 (*m*), 1536 (*m*), 1498 (*m*), 1426 (*w*), 1400 (*m*), 1348 (*s*), 1277 (*m*), 1246 (*m*), 1191 (*m*), 1151 (*m*), 1132 (*w*), 1086 (*w*), 1027 (*w*), 972 (*w*), 951 (*w*), 926 (*w*), 868 (*w*), 824 (*w*), 767 (*w*), 719 (*w*), 700 (*w*), 669 (*w*). Elemental analysis (CHN) calculated (%) for Ca_3_PQQ_2_·11H_2_O or C_28_H_28_Ca_3_N_4_O_27_: C 34.57, H 2.90, N 5.76; found: C 34.30, H 3.20, N 6.06. Crystals were picked out of the reaction mixture and then dried for 1 d on filter paper prior to elemental analysis.

### Refinement   

Crystal data, data collection and structure refinement details are summarized in Table 1 ▸. Four reflections have been omitted from the refinement. Three of them are hidden by the beam stop and show no intensity. A further omitted reflection of higher order (090) has a significantly higher *F*_o_^2^ (63.72) com­pared to its *F*_c_^2^ (1.09). This behaviour is observed quite often for reflections of higher order when multigraded X-ray mirrors are used as monochromators. All C-bound H atoms have been calculated in ideal geometry riding on their parent atoms, while the O- and N-bound H atoms were refined freely. Full details of the refinement strategy can be found in the embedded instruction file in the CIF.

## Results and discussion   

### Investigation of PQQ–Ca com­plexation   

In our previous article, PQQ–metal com­plexes were reported with the trivalent lanthanides La^3+^, Eu^3+^ and Lu^3+^, and with Ca^2+^ (Lumpe & Daumann, 2019 ▸). Regardless of the excess of added metal salt, 1:1 com­plexes were identified by elemental analysis. While no further structural information could be provided in that study, we were recently able to verify the proposed stoichiometry by the crystal structure of an Eu–PQQ com­plex with the net formula Eu_2_PQQ_2_·12H_2_O (Lumpe *et al.*, 2020 ▸). The Eu^3+^ ion is coordinated by PQQ in the same fashion as in MDH, with participation of N2, O4 and O5, in addition to the participation of O1 (Fig. 2 ▸). The latter residue is not utilized in the enzyme for metal coordination.

From a similar experimental approach using Ca^2+^ instead of Eu^3+^, single crystals suitable for X-ray analysis were grown over a period of several days. Fig. 3 ▸ illustrates the com­position of the asymmetric unit: the charges of two triply deprotonated PQQ units are balanced by three Ca^2+^ ions supplemented by 13 water mol­ecules. The structural motif depicted in Fig. 2 ▸ – the formation of binuclear units by means of two PQQ ligands acting as linkers between the metal centres – is realized in Ca_3_PQQ_2_·13H_2_O in a com­parable fashion for two of the three Ca ions (Ca1 and Ca3). Ca1 is coordinated by PQQ in a similar fashion to Eu; however, the coordination sphere is com­pleted by a carboxyl­ate group of a nearby pyridine moiety of PQQ (instead of a carboxyl­ate of a pyrrole ring). Ca3, on the other hand, uses the same pocket and residues as Eu; however, this inter­action is assisted by a hydrogen bond of a Ca3-bound water mol­ecule to the carboxyl­ate group of the pyrrole ring (Fig. 3 ▸ *b*, green arrows). In the structure, these two types of alternating Ca1 and Ca3 units are connected *via* Ca1 into strands along [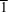
11]. The charge of the Ca_2_PQQ_2_ unit is balanced by Ca2, which is coordinated solely by water mol­ecules and carboxyl­ate groups, however, never in the biologically relevant ONO pocket of PQQ. All N—H and O—H donor groups are involved in classical hydrogen bonds with either carboxyl­ate groups, keto groups or water mol­ecules, acting as acceptors establishing a three-dimensional network (see Table 2 ▸ for hydrogen-bond details).

Inter­estingly, while the elemental analysis of the initially precipitated (amorphous) solid showed a 1:1 Ca–PQQ stoichiometry (Lumpe & Daumann, 2019 ▸), the present structure from slowly crystallized material reveals a network of three different Ca^2+^ ions and two differently-coordinated PQQ anionic ligands, resulting in a 3:2 stoichiometry. Also, in the Ca–PQQ structure, both PQQ mol­ecules coordinate in the same fashion as in the MDH enzyme (Fig. 1 ▸), in addition to the participation of several carboxylate groups. One of the PQQ ligands coordinates to calcium with the participation of all three carboxylate groups: Ca1 *via* O8, N2, O5 and O4, and Ca2 *via* O1 and O2 in a bidentate manner. The second PQQ mol­ecule coordinates with only two of the three carboxylate groups and coordinates Ca1 with O14, Ca2 with O15 and Ca3 with N4, O12 and O13. In total, 13 water mol­ecules are present in the crystal structure, of which 11 directly coordinate to atoms Ca1–Ca3 and two water mol­ecules (O28 and O29) have no direct coordination partners. Inter­estingly, elemental analysis of the dried crystalline material fits best to only 11 water mol­ecules, most likely due to the disappearance of the two noncoordinating water mol­ecules during the drying process. Ca1 and Ca2 show penta­gonal–bipyramidal geometries, with coordination numbers (CNs) of 7 and Ca3 shows a distorted geometry with a CN of 8. All metal-to-ligand bond lengths and angles of Ca_3_PQQ_2_ are given in Table 3 ▸, in addition to the values for Eu_2_PQQ_2_. The known PQQ–water adduct (diol in C5 position), which is formed to some extent in aqueous solution (Dekker *et al.*, 1982 ▸), is not present in the com­plex, and this is in line with all known crystal structures of PQQ, to the best of our knowledge.

In the Eu_2_PQQ_2_ com­plex, the Eu ions are coordinated in a similar fashion by PQQ. The bonds to Eu are up to 0.141 Å longer than to Ca1 and Ca3. The CN of Eu in the com­plex is 9, which corresponds to an ionic radius of 1.12 Å according to Shannon (1976 ▸), while the ionic radius of Ca is 1.06 Å for a CN of 7 and 1.12 Å for a CN of 8. Therefore, the larger bond lengths to Eu can hardly be explained by different ionic radii, which are overall similar, but by differences in the CNs and different participation in coordination of a second PQQ mol­ecule.

**Figure 4:**
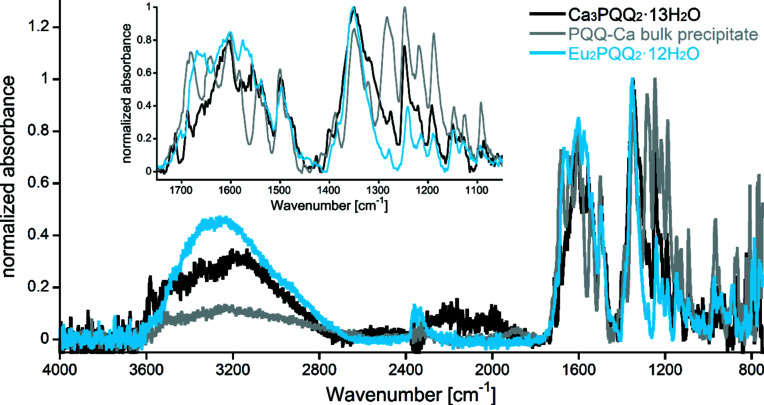
Normalized IR absorption spectra of the Ca_3_PQQ_2_ com­plex in black, the 1:1 Ca–PQQ precipitate in grey and the Eu_2_PQQ_2_ com­plex in blue. Inset: close-up of the PQQ-related IR absorption peaks.

The IR spectra of the precipitated Ca–PQQ amorphous solid, Eu_2_PQQ_2_ and Ca_3_PQQ_2_ crystals were recorded and com­pared (Fig. 4 ▸). The spectra can be roughly divided into two areas. While PQQ C=O stretching vibrations of the carboxylate and quinone groups absorb in the range 1750–1600 cm^−1^ (Zhejiang Hisun Pharmaceutical Co. Ltd, 2020 ▸), the peaks with smaller wavenumbers are largely related to PQQ lattice vibrations. While the heights of the large absorption bands in the range 3600–2600 cm^−1^ are a direct result of the different amounts and coordination modes of cocrystallized water, the differences in the area 1750–1550 cm^−1^ further indicate the different coordination modes already depicted in the crystal structures.

## Conclusion   

We present here the first crystal structure of PQQ with the biologically relevant metal ion calcium. The com­plex consists of PQQ and the metal ion alone, unlike previously reported structures with other metal ions. Those com­plexes often needed additional co-ligands, which limited the use of the structures for com­parison with the biologically active site. However, in particular, the use of methyl­ated PQQMe_3_ (with all three carboxyl groups esterified) prevented participation of (nonbiogenic) carboxyl groups in com­plexation. This is not the case in the presented structure, where calcium is coordinated by PQQ in the same pocket as in MDH, in addition to further carboxyl-group participation, spanning a three-dimensional coordination network. However, considering the few crystal structures of PQQ com­plexes reported over the years, we are confident that the presented structure will help to better explain the coordination behaviour of PQQ outside the MDH enzyme and help guide the design of mononuclear model com­plexes for these fascinating enzymes.

## Supplementary Material

Crystal structure: contains datablock(s) I, global. DOI: 10.1107/S2053229620014278/yo3076sup1.cif

Structure factors: contains datablock(s) I. DOI: 10.1107/S2053229620014278/yo3076Isup2.hkl

CCDC reference: 2019890

## Figures and Tables

**Table 1:** Experimental details

Crystal data	
Chemical formula	[Ca_3_(C_14_H_3_N_2_O_8_)_2_(H_2_O)_11_]·2H_2_O
*M*_r_	1008.81
Crystal system, space group	Triclinic, *P* 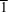
Temperature (K)	109
*a*, *b*, *c* (Å)	6.9363 (3), 15.9791 (7), 16.9786 (7)
α, β, γ (°)	90.844 (1), 93.106 (1), 98.296 (2)
*V* (Å^3^)	1858.93 (14)
*Z*	2
Radiation type	Mo *K*α
μ (mm^−1^)	0.56
Crystal size (mm)	0.10 × 0.02 × 0.01

Data collection	
Diffractometer	Bruker D8 Venture TXS
Absorption correction	Multi-scan (*SADABS*; Bruker, 2016 ▸)
*T*_min_, *T*_max_	0.88, 0.99
No. of measured, independent and observed [*I* > 2σ(*I*)] reflections	33048, 8166, 7023
*R*_int_	0.043
(sin θ/λ)_max_ (Å^−1^)	0.641

Refinement	
*R*[*F* ^2^ > 2σ(*F* ^2^)], *wR*(*F* ^2^), *S*	0.031, 0.072, 1.04
No. of reflections	8166
No. of parameters	689
H-atom treatment	H atoms treated by a mixture of independent and constrained refinement
Δρ_max_, Δρ_min_ (e Å^−3^)	0.39, −0.28

Computer programs: *APEX3* (Bruker, 2016 ▸), *SAINT* (Bruker, 2017 ▸), *SHELXT2014* (Sheldrick, 2015 ▸ *a*), *SHELXL2018* (Sheldrick, 2015 ▸ *b*) and *ORTEP-3* (Farrugia, 2012 ▸).

**Table 2:** Hydrogen-bond geometry (Å, °)

*D*—H⋯*A*	*D*—H	H⋯*A*	*D*⋯*A*	*D*—H⋯*A*
N1—H2⋯O8	0.86 (2)	2.01 (2)	2.7232 (19)	139.6 (19)
N3—H4⋯O16	0.86 (2)	1.83 (2)	2.6163 (19)	151 (2)
O17—H171⋯O10^iii^	0.85 (3)	1.91 (3)	2.7543 (18)	170 (3)
O17—H172⋯O1	0.86 (3)	2.08 (3)	2.9287 (19)	170 (2)
O18—H181⋯O28	0.88 (3)	1.82 (3)	2.681 (2)	166 (2)
O18—H182⋯O1^iv^	0.84 (3)	1.97 (3)	2.8107 (19)	176 (3)
O19—H191⋯O20^ii^	0.81 (3)	2.10 (3)	2.8789 (19)	161 (3)
O19—H192⋯O5^i^	0.85 (3)	1.89 (3)	2.7350 (18)	174 (3)
O20—H201⋯O11^v^	0.82 (3)	2.00 (3)	2.8221 (18)	173 (3)
O20—H202⋯O2^vi^	0.84 (3)	1.94 (3)	2.7620 (18)	169 (3)
O21—H211⋯O29^iii^	0.80 (3)	2.05 (3)	2.845 (2)	173 (3)
O21—H212⋯O16	0.85 (3)	1.90 (3)	2.7320 (18)	163 (3)
O22—H221⋯O19^vi^	0.76 (3)	2.26 (3)	2.958 (2)	152 (3)
O22—H222⋯O6^vii^	0.87 (3)	1.83 (3)	2.6985 (19)	175 (3)
O23—H231⋯O3^viii^	0.76 (3)	2.10 (3)	2.8398 (19)	164 (3)
O23—H232⋯O9^iii^	0.83 (3)	1.90 (3)	2.7217 (19)	169 (3)
O24—H241⋯O7^ix^	0.81 (3)	2.03 (3)	2.8040 (19)	163 (3)
O24—H242⋯O29	0.82 (3)	1.93 (3)	2.750 (2)	172 (3)
O25—H251⋯O13^ix^	0.85 (3)	2.02 (3)	2.8578 (18)	168 (3)
O25—H252⋯O3^viii^	0.81 (3)	2.41 (3)	3.0258 (19)	133 (2)
O25—H252⋯O4^viii^	0.81 (3)	2.28 (3)	3.0405 (18)	155 (3)
O26—H261⋯O27^x^	0.86 (3)	2.11 (3)	2.9018 (19)	154 (3)
O26—H262⋯O13^ix^	0.80 (3)	2.06 (3)	2.8506 (19)	170 (3)
O27—H271⋯O25^iv^	0.85 (3)	2.08 (3)	2.9022 (19)	164 (3)
O27—H272⋯O9^v^	0.83 (3)	1.99 (3)	2.8125 (18)	171 (2)
O28—H281⋯O10^v^	0.89 (4)	1.88 (4)	2.721 (2)	159 (3)
O28—H282⋯O26^x^	0.83 (4)	2.59 (3)	3.098 (2)	121 (3)
O29—H291⋯O6^ix^	0.84 (3)	1.85 (3)	2.6582 (19)	160 (3)
O29—H292⋯O11	0.79 (3)	2.27 (3)	3.021 (2)	159 (3)
O29—H292⋯O12	0.79 (3)	2.40 (3)	2.7789 (19)	110 (2)

Symmetry codes: (i) 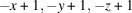; (ii) 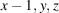; (iii) 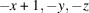; (iv) 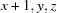; (v) 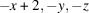; (vi) 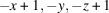; (vii) 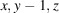; (viii) 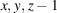; (ix) 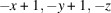; (x) 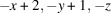.

**Table 3:** Selected bond lengths (Å) of the Ca_3_PQQ_2_·13H_2_O com­plex in com­parison with the previously reported Eu_2_PQQ_2_·12H_2_O structure For symmetry data for Eu_2_PQQ_2_·12H_2_O, see Lumpe *et al.* (2020 ▸).

Ca_3_PQQ_2_·13H_2_O		Eu_2_PQQ_2_·12H_2_O	
Ca1^i^—O4	2.5928 (12)	Eu1—O4	2.584 (2)
Ca1^i^—N2	2.5069 (14)	Eu1—N2	2.648 (2)
Ca1^i^—O5	2.3784 (12)	Eu1—O5	2.440 (2)
Ca1—O14	2.2514 (12)	Eu1—O1	2.409 (2)
Ca1—O8	2.3137 (12)	Eu1—O_water_ (5 bonds)	2.389 (2)–2.464 (2)
Ca1—O17	2.3694 (13)		
Ca1—O18	2.3145 (14)	Ca3—O12	2.5703 (12)
Ca2^ii^—O1	2.4812 (12)	Ca3—N4	2.5460 (14)
Ca2^ii^—O2	2.5279 (12)	Ca3—O13	2.3963 (12)
Ca2—O15	2.3522 (12)	Ca3—O23	2.4362 (14)
Ca2—O19	2.3894 (14)	Ca3—O24	2.3607 (13)
Ca2—O20	2.4382 (13)	Ca3—O25	2.5787 (14)
Ca2—O21	2.3824 (14)	Ca3—O26	2.3962 (14)
Ca2—O22	2.3383 (14)	Ca3—O27	2.4924 (14)

Symmetry codes: (i) −*x* + 1, −*y* + 1, −*z* + 1; (ii) *x* − 1, *y*, *z*.
